# Cardiovascular disease risk prediction models for people living with HIV in Sub-Saharan Africa: A scoping review protocol

**DOI:** 10.1371/journal.pone.0352716

**Published:** 2026-11-06

**Authors:** Nchidzi Ntiyani, Gobopamang Letamo, Mpho Keetile, Mpho Disang

**Affiliations:** Department of Statistics, Demography & Population Studies, University of Botswana, Botswana, Southern Africa; Keimyung University, KOREA, REPUBLIC OF

## Abstract

**Background:**

The landscape of Human Immunodeficiency Virus (HIV) care has dramatically shifted with the advent of effective antiretroviral therapy (ART), transforming HIV from a rapidly fatal illness into a chronic, manageable disease. This success, however, has unveiled new challenges, primarily the increased prevalence of non-AIDS-related comorbidities, with non-communicable diseases (NCDs) such as cardiovascular disease (CVD) emerging as a critical concern for people living with HIV (PLWH). CVD has now become a leading cause of morbidity and mortality in this population, and its risk assessment is imperative. However, there is scanty data on CVD risk prediction models developed and/or applied among PLWH in sub-Saharan Africa (SSA). Therefore, this planned scoping review aims to systematically map existing literature on CVD risk prediction models developed and/or used among PLWH in SSA. The review will identify the models that have been developed, used, and validated in the region.

**Methods:**

The scoping review protocol will follow an established framework for conducting scoping reviews. The study will employ the methodological approaches by Arksey and O’Malley and its recent advances, the Joanna Briggs Institute, and the Preferred Reporting Items for Systematic Reviews and Meta-Analysis Protocols. The objective of the scoping review will be achieved through a comprehensive published literature search from 01 January 2000 to 03 June 2026 across five selected databases: PubMed, Scopus, Web of Science, Google Scholar, and CINAHL, based on a predefined strategy. Inclusion criteria will focus on studies written in English that develop, validate, or apply CVD risk prediction models among PLWH in SSA. The screening of search results and data extraction will be conducted by two independent reviewers.

**Expected outcomes and conclusions:**

The review will synthesize and summarize the existing literature on CVD risk prediction developed and used among PLWH in SSA, identify knowledge gaps, and provide insights to inform the development of a novel CVD risk prediction model specific to PLWH in SSA. Registration: Open Science Framework https://doi.org/10.17605/OSF.IO/XVFUA

## Introduction

Human Immunodeficiency Virus (HIV) continues to be a global public health problem. According to the World Health Organization (WHO), approximately 39.0 million people were living with HIV worldwide at the end of 2022, and an estimated 25.6 million resided in Africa [1]. Sub-Saharan Africa (SSA) is the epicenter of HIV, bearing the heaviest burden by accounting for 67% of all people globally [2]. Historically, HIV has been synonymous with high mortality due to immunosuppression and vulnerability to opportunistic infections. However, the advent of antiretroviral therapy (ART) has marked a paradigm shift in the understanding of HIV, from a progressive fatal disease to a chronic progressive disease. Approximately 29.8 million people living with HIV (PLWH) were receiving ART globally, with 70% of those from the African region in 2022 [3]. With increased access to ART, PLWH are now living longer, with shifting morbidity profiles toward noncommunicable diseases, particularly cardiovascular disease (CVD).

CVD is the leading cause of death globally, accounting for an estimated 17.9 million deaths annually, representing 32% of all global deaths [4]. Traditionally considered distinct epidemics, HIV and CVD increasingly intersect, with evidence showing that PLWH have a 2-fold risk of CVD than people who are HIV negative [5]. HIV infection is associated with chronic inflammation and immune dysregulation, while certain ART agents are associated with cardiometabolic changes that elevate the risk of CVD [6–8]. As a result of the existence of these two synergistic co-epidemics, the assessment and prediction of CVD risk have become an important aspect of HIV management. CVD risk assessment is important for CVD prevention and enables medical practitioners and their patients to make informed decisions. Many CVD risk assessment and prediction tools for estimating the probability of developing CVD, such as the Framingham Risk Score (FRS), Pooled Cohort Equations of the American Heart Society/American score (PCE), Systematic COronary Risk Evaluation (SCORE), and the World Health Organization cardiovascular disease risk charts have been developed [9–12]. These risk prediction models were developed for use among the general population, using populations from high-income countries in Europe and North America, and do not include HIV-specific factors. HIV-specific CVD prediction models such as the Data collection on Adverse Effects of Anti-HIV Drugs Study risk equation (D:A:D) have also been developed in developed countries [13]. Despite the plethora of CVD risk prediction models, they have been found to inaccurately predict CVD risk among PLWH [14,15]. The suboptimal performance of these risk prediction models among PLWH has been attributed to multiple factors, including the population characteristics and HIV-related factors [14]. The scoping review will therefore allow us to identify and synthesize existing CVD risk prediction models developed or applied among PLWH in SSA. The scoping review aims to describe the features of the identified risk prediction models. It is anticipated that the findings of this scoping review will provide a basis for future research and provide insight for improving CVD risk prediction among PLWH in SSA.

## Materials and methods

### Study design

We will systematically conduct a scoping review of peer-reviewed literature to map the evidence on CVD risk prediction models developed and/or applied among PLWH in SSA. The proposed scoping review will be guided by the methodological framework developed by [16]. This framework entails following the outlined five steps: (i) identifying the research question, (ii) identifying relevant studies, (iii)selection of eligible studies, (iv) charting the data, and (v) collating, summarising, and reporting the results. The scoping review protocol will also be guided by the methodology developed by the Joanna Briggs Institute (JBI) [17], which is congruent with the Preferred Reporting Items for Systematic Reviews and Meta-Analysis Protocols (PRISMA-P) (S1 File) [18]. The review will apply the PRISMA extension for scoping reviews (PRISMA-ScR) to present the results.

We aim to answer the following questions:

- What cardiovascular disease risk prediction models were developed or used to predict CVD among PLWH in SSA?
- Which factors/predictors and outcomes were included in the model?
- How did the model perform in predicting CVD?

### Identifying relevant studies

A search of peer-reviewed literature will be conducted. The search will be conducted across five selected databases, being PubMed, Scopus, Web of Science, Google Scholar, and CINAHL, using a predefined strategy. The search strategy will combine search terms relating to HIV, cardiovascular disease, risk prediction models, and sub-Saharan Africa. Synonymous terms within each concept will be combined using OR, while the concepts will be combined using AND. Studies from January 2000 to 03 June 2026 that have keywords or Medical Subject Headings (MeSH) related to CVD prediction models and HIV will be selected. The search may be updated to ensure the review includes the most recent literature at the time of publication. The search terms, Boolean operators, and complete PubMed search strategy are provided in (S2 File). Articles will be retrieved from each database and imported into EndNote.

### Selection of eligible studies

The study will use the Population-Concept-Context (PCC) framework to determine the eligibility criteria (Table 1). This framework will guide the title and abstract screening as well as ensuring the content of the included studies is relevant to the research question.

**Table 1:** PCC framework for eligibility criteria.

Criteria	Determinant
Population	HIV positive participants aged 18 and above
Concept	Articles focusing on CVD risk prediction models developed or used
Context	Sub-Saharan Africa

#### Inclusion criteria.

- Focus on the HIV-positive population aged 18 and above. Studies including both HIV-positive and HIV-negative participants will be eligible only when they performed separate stratified analyses for individuals who are HIV-positive and HIV-negative and reported results separately.
- Report on CVD risk prediction models developed, validated, or applied. Eligible cardiovascular outcomes will include myocardial infarction, coronary heart disease, angina, stroke, peripheral arterial disease, heart failure, cardiovascular death, or other clinically defined cardiovascular events. Studies reporting composite cardiovascular outcomes will also be eligible, provided that the composite includes clinically relevant cardiovascular events.
- Include participants in SSA.
- Published from 01/01/2000 to 03/06/2026.
- Quantitative prospective and retrospective cohort studies reporting observed cardiovascular events, and cross-sectional studies reporting estimated cardiovascular risk scores will be eligible.

#### Exclusion criteria.

- Studies that do not include people who are HIV positive, younger than 18, and are not from SSA.
- Studies reporting only individual cardiovascular risk factors or their prevalence, without applying or evaluating a cardiovascular disease risk prediction model or reporting estimated cardiovascular risk, will be excluded.
- Studies where the full text could not be obtained.
- Studies not in English.
- Studies published before 01/01/2000.
- Correspondences, commentaries, and editorials will be excluded.

### Study selection

We will use a PRISMA flow chart to summarize the study findings. All the studies meeting the inclusion criteria will be exported to EndNote 21 software for data management and referencing. Two independent reviewers will conduct the title and abstract screening to select studies related to our PCC as described above. All the studies that do not address the study’s research questions will be excluded, including all duplicates. Full-text articles will be retrieved for all the titles and abstracts that potentially meet the inclusion criteria. Full-text articles that do not meet the inclusion criteria will be excluded, and reasons for the exclusion will be provided. In instances where there is difficulty in finding articles, the researchers will seek assistance from the University of Botswana’s library services. Authors will also be contacted, and full-text copies of articles that are not attainable within the University of Botswana will be requested. If the reviewers cannot reach a consensus regarding some articles during screening, a third reviewer will be requested to resolve the issue. Full articles of the eligible studies will then be used for data extraction. The results of the search and the study inclusion process will be reported in full in the final scoping review and presented in a PRISMA flow chart.

### Data extraction and charting

The data extraction process will utilize the data extraction tool adapted from the JBI standardized data extraction form, aligned with the research question. The following information will be extracted from each included study: publication details (author(s), year of publication, country and setting); aims or study purpose; study characteristics (study design, study population and sample size); characteristics of the CVD risk prediction model (model name/type and whether the model was developed, validated, or applied); predictors included in the model; outcome(s) and their definitions; prediction period (e.g., 5-year or 10-year risk); and reported measures of model performance, including discrimination and calibration measures, where available. For studies reporting composite cardiovascular outcomes, the individual components of the composite outcome will be extracted and recorded.

The full data extraction will be independently conducted by two reviewers, with discrepancies resolved by discussion or a third reviewer. Information that is not reported in an included study will be recorded as “not reported.”

### Collating, summarising, and reporting the results

The analysis will be descriptive and narrative, consistent with the aims of a scoping review. Results will be synthesized through frequency counts, cross-tabulations, and trend descriptions to map the models used, the predictors most frequently included, the geographical distribution of studies, and the performance of different models.

Specifically, the synthesis will focus on the types of cardiovascular risk prediction models developed or applied in SSA, including the predictors used in model development, the geographical coverage of the models, and the reported model performance.

### Study status and timeline

At the time of initial protocol submission, only the PUBMED database search had been conducted. The comprehensive search across other databases has since been completed, with the records imported into EndNote. Title and abstract screening of records was completed on the 25th of September 2026. Full-text review and data extraction are underway and anticipated to be completed by 31st of October 2026. Data synthesis, interpretation, and final manuscript preparation are expected to be completed by the end of November 2026, with publication of the review findings anticipated thereafter. At the time of submission, no data analysis or synthesis has been undertaken, and no results have been generated.

## Discussion

This scoping review maps and synthesizes available literature on CVD risk prediction models developed, validated, or applied and used among PLWH in SSA. SSA is the epicenter of HIV [2]. PLWH have a heightened risk of CVD, which is reported to be 2-fold higher than those without HIV [4,19,20]. The ability to predict this risk before CVD occurs is very important for timely risk stratification and interventions and preventing CVD complications such as disability and mortality. However, the extent of CVD models developed, validated or used and their performances in PLWH in SSA remains unclear. CVD risk assessment and prediction among PLWH in SSA is largely unknown. Therefore, by mapping existing evidence, this scoping review will provide a region-specific review, identify gaps, and improve understanding of CVD risk stratification and prevention among PLWH in SSA.

The scoping review will particularly provide evidence on the reported performance of the models in the context of SSA. It will summarize evidence on calibration and discrimination of the models and identify if the models were developed specifically for PLWH. Evidence suggests that models that are externally derived can demonstrate different levels of calibration or discrimination when applied to populations they were not derived from, primarily due to differences in disease epidemiology and population characteristics [21]. By systematically mapping which models have been developed, applied, or validated in this setting, and synthesizing available evidence on their performance and applicability, this review is expected to determine whether existing tools are adequate for the region or whether there is a need for developing context-specific, SSA CVD prediction models.

Furthermore, the review will also summarize the predictors and outcomes used in the models, providing evidence on the applicability of the models. When developing risk prediction models, it is important to consider the users of the model. The usability of a risk prediction model is influenced among others by the availability of predictors used in the model. Therefore, it is imperative that the predictors used in the model are those that are routinely collected in clinical settings to ease the clinical implementation of the model [22].

This review will extend beyond existing global reviews of CVD risk prediction models in PLWH, which have not specifically interrogated model performance and applicability within the SSA context. This review will have a specific focus on CVD risk prediction models among PLWH in SSA, providing a population- and region-specific synthesis of the available evidence. It will consider models that have been developed, validated, or applied in this population, allowing a broader assessment of the current evidence base. Several limitations of this scoping review should however, be acknowledged. The selection of studies published in the English language only may lead to exclusion of relevant data from studies published in other languages. There may also be a limited number of published studies on CVD risk prediction models developed in SSA. However, such a finding would itself be important, as it would underscore the need for development of context-specific CVD risk prediction models in the region.

## Supporting information

S1 File PRISMA-P 2015 checklist. (DOCX)

S2 File PubMed search strategy. (DOCX)

## Abbreviations

ART: Antiretroviral therapy
CVD: Cardiovascular disease
HIV: Human immunodeficiency virus
JBI: Joanna Briggs Institute
NCDs: Non-communicable diseases
PCC: Population-Concept-Context
PLWH: People living with HIV
PRISMA-P: Preferred Reporting Items for Systematic and Meta-Analysis Protocol
PRISMA-ScR: Preferred Reporting Items for Systematic Reviews and Meta-Analysis extension for Scoping Reviews
SSA: Sub-Saharan Africa
