# Supplementary material for: Cardiovascular disease risk prediction models for people living with HIV in Sub-Saharan Africa: A scoping review protocol
Source: PLoS One. 2026 Nov 6;21(11):e0352716. doi: 10.1371/journal.pone.0352716 (PMC13743019; doi:10.1371/journal.pone.0352716)
Supplement: Supplementary file 1 [file pone.0352716.s001.docx]

**PRISMA-P (Preferred Reporting Items for Systematic review and Meta-Analysis Protocols) 2015 checklist: recommended items to address in a systematic review protocol***

| Section and topic | Item No | Checklist item |
| --- | --- | --- |
| ADMINISTRATIVE INFORMATION | | |
| Title: |  |  |
| Identification | 1a | Cardiovascular disease risk prediction models for people living with HIV in Sub-Saharan Africa: A scoping review protocol |
| Update | 1b |  |
| Registration | 2 | This scoping review protocol has been submitted to Open Science Framework with a DOI of <https://doi.org/10.17605/OSF.IO/XVFUA> |
| Authors: |  |  |
| Contact | 3a | *Nchidzi Ntiyani^1*^, Gobopamang Letamo^1^, Mpho Keetile^1^, Mpho Disang^1^*  *^1^Department of Statistics, Demography & Population Studies, University of Botswana, Botswana*  *Corresponding author  Email: nntiyani@gmail.com |
| Contributions | 3b | Conceptualization: NN, GL, MD  Formal analysis: NN, MD  Methodology: NN, MD  Supervision: GL, MK & MD  Validation: NN, GL, MK, MD  Writing – original draft: NN  Writing – review and editing: GL, MK & MD |
| Amendments | 4 | N/A |
| Support: |  |  |
| Sources | 5a | This review did not get any form of external support |
| Sponsor | 5b | This review had no sponsor |
| Role of sponsor or funder | 5c | N/A |
| INTRODUCTION | | |
| Rationale | 6 | Many cardiovascular risk prediction models used to assess and classify cardiovascular risk were developed in high income countries and evidence show that they could not accurately predict cardiovascular risk among people living with HIV (PLWH). The suboptimal performance of these risk prediction models among PLWH has been attributed to multiple factors, including the population characteristics and HIV-related factors (Achhra et al., 2021). Despite the high burden of HIV in sub-Saharan Africa, it remains unclear which CVD risk prediction models have been developed, validated, or applied among PLWH in the region and what their characteristics and performance are. The scoping review will highlight evidence gaps and provide a foundation for future research aimed at developing and validating contextually relevant HIV-specific CVD risk prediction models in the region. |
| Objectives | 7 | The scoping review aims to describe the features of the identified risk prediction models. It is anticipated that the findings of this scoping review will provide a basis for future research and provide insight for improving CVD risk prediction among PLWH in sub-Saharan Africa. |
| METHODS | | |
| Eligibility criteria | 8 | The study will use the Population-Concept-Context (PCC) framework to determine the eligibility criteria.  Population  The review will include studies that develop, validate or apply cardiovascular risk prediction among people living with HIV aged 18 years and above. This will include studies involving the general population but also involving PLWH.  Concept  The review will consider studies which develop, validate or apply CVD risk prediction published from 01/01/2000 to 03/06/2026. Peer reviewed quantitative prospective, retrospective and cross-sectional studies will be included. Only studies written in English will be considered for the review.  Context  The review will consider studies conducted in sub-Saharan Africa. |
| Information sources | 9 | The review will conduct a search across selected databases, being PubMed, Scopus, Web of Science, Google Scholar, and CINAHL, using a predefined strategy. Studies from January 2000 to 03 June 2026 that have the keywords or Medical Subject Headings (MeSH) related to CVD prediction models and HIV will be selected. The search may be updated to ensure the most recent literature is included in the review at time of publication. |
| Search strategy | 10 | The draft search strategy is attached |
| Study records: |  |  |
| Data management | 11a | After the search, all identified studies will be collated and uploaded into Endnote 21 and all records of duplicates will be removed. |
| Selection process | 11b | Two independent reviewers will conduct the title and abstract screening to select studies related to our population, concept, and context (PCC). All the studies that do not address the study’s research questions will be excluded, including all duplicates. Full-text articles will be retrieved for all the titles and abstracts that potentially meet the inclusion criteria. Full-text articles that do not meet the inclusion criteria will be excluded, and reasons for the exclusion will be provided. In instances where there is difficulty in finding articles, the researchers will seek assistance from the University of Botswana's library services. Authors will also be contacted, and full-text copies of articles that are not attainable within the University of Botswana will be requested. If the reviewers cannot reach a consensus regarding some articles during screening, a third reviewer will be requested to resolve the issue. Full articles of the eligible studies will then be used for data extraction. The results of the search and the study inclusion process will be reported in full in the final scoping review and presented in a PRISMA flow chart. |
| Data collection process | 11c | The data extraction process will utilize the data extraction tool adapted from the Joanna Briggs Institute (JBI) standardized data extraction form, aligned with the research question. This tool includes the following: the publication title, author(s), year of publication, country and setting, aims, population characteristics, methodology, cardiovascular risk prediction models developed or applied, predictors and outcomes used in the models, and model performance. The full data extraction will be independently conducted by two reviewers, with discrepancies resolved by discussion or third reviewer. |
| Data items | 12 | List and define all variables for which data will be sought (such as PICO items, funding sources), any pre-planned data assumptions and simplifications |
| Outcomes and prioritization | 13 | List and define all outcomes for which data will be sought, including prioritization of main and additional outcomes, with rationale |
| Risk of bias in individual studies | 14 | N/A |
| Data synthesis | 15a | The analysis will be descriptive and narrative, consistent with the aims of a scoping review. Results will be synthesized through frequency counts, cross-tabulations, and trend descriptions to map the models used, the predictors most frequently included, the geographical distribution of studies, and the performance of different models. |
|  | 15b | N/A |
|  | 15c | N/A |
|  | 15d | A narrative summary will be used to describe the results based on the research questions which guided the review which can guide future development. We will publish the outcome of this scoping review in a peer-reviewed journal as well as present at national, regional or international conferences where possible. |
| Meta-bias(es) | 16 | N/A |
| Confidence in cumulative evidence | 17 | N/A |

*** It is strongly recommended that this checklist be read in conjunction with the PRISMA-P Explanation and Elaboration (cite when available) for important clarification on the items. Amendments to a review protocol should be tracked and dated. The copyright for PRISMA-P (including checklist) is held by the PRISMA-P Group and is distributed under a Creative Commons Attribution Licence 4.0.**

*From: Shamseer L, Moher D, Clarke M, Ghersi D, Liberati A, Petticrew M, Shekelle P, Stewart L, PRISMA-P Group. Preferred reporting items for systematic review and meta-analysis protocols (PRISMA-P) 2015: elaboration and explanation. BMJ. 2015 Jan 2;349(jan02 1):g7647.*
