# Supplementary material for: Cardiovascular disease risk prediction models for people living with HIV in Sub-Saharan Africa: A scoping review protocol
Source: PLoS One. 2026 Nov 6;21(11):e0352716. doi: 10.1371/journal.pone.0352716 (PMC13743019; doi:10.1371/journal.pone.0352716)
Supplement: Supplementary file 2 [file pone.0352716.s002.docx]

**Cardiovascular disease prediction models for people living with HIV in Sub-Saharan Africa: A scoping review protocol**

**PubMed**

**Date of Search:** 03 June 2026

**Number of Results:** 409

“Cardiovascular Diseases”[Mesh] OR CVD[tiab] OR “Cardiovascular Disease”[tiab] OR “Disease, Cardiovascular”[tiab] OR “Cardiac Events”[tiab] OR “Cardiac Event”[tiab] OR “Event, Cardiac”[tiab] OR “Adverse Cardiac Event”[tiab] OR “Adverse Cardiac Events”[tiab] OR “Cardiac Event, Adverse”[tiab] OR “Cardiac Events, Adverse”[tiab] OR “Major Adverse Cardiac Events”[tiab] OR “Coronary Heart Disease”[tiab] OR CHD[tiab] OR “Ischemic Diseases”[tiab] OR “Coronary Artery Disease”[tiab] OR CAD[tiab] OR “Myocardial Infarction”[tiab] OR “Heart Attack”[tiab] OR “Angina Pectoris”[tiab] OR “Coronary Artery Spasm”[tiab] OR “Cerebrovascular Disease”[tiab] OR Stroke[tiab] OR Ischemic[tiab] OR “Transient Ischemic Attack”[tiab] OR TIA[tiab] OR “Cerebral Aneurysm”[tiab] OR “Heart Failure”[tiab] OR Cardiomyopathy[tiab] OR “Congestive Heart Failure”[tiab] OR Cardiomyopathy[tiab] OR Arrhythmias[tiab] OR “Heart Rhythm Disorders”[tiab] OR “Atrial Fibrillation”[tiab] OR AFib[tiab] OR Bradycardia[tiab] OR “Ventricular Tachycardia”[tiab] OR Fibrillation[tiab] OR “Peripheral Vascular Disease”[tiab] OR PVD[tiab] OR “Peripheral Artery Disease”[tiab] OR PAD[tiab] OR “Deep Vein Thrombosis”[tiab] OR DVT[tiab] OR “Pulmonary Embolism”[tiab] OR PE[tiab] OR “Varicose Veins”[tiab] OR “Valvular Heart Disease”[tiab] OR “Structural Heart Disease”[tiab] OR “Heart Valve Disease”[tiab] OR “Congenital Heart Disease”[tiab] OR “Congenital Heart Disease”[tiab] OR “Aortic Diseases”[tiab] OR “Pericardial Diseases”[tiab] OR “Aortic Aneurysm”[tiab] OR “Aortic Dissection”[tiab] OR Pericarditis[tiab] OR “Hypertensive Heart Diseases”[tiab] OR “Inflammatory Heart Diseases”[tiab] OR “Hypertensive Heart Disease”[tiab] OR Endocarditis[tiab] OR Myocarditis[tiab] AND “Risk Prediction”[tiab] OR “Risk Predictions”[tiab] OR "Risk Prediction Models"[tiab] OR "Risk Prediction Model"[tiab] OR “Risk Assessment”[tiab] OR “Risk Assessments”[tiab] OR “Risk Factor”[tiab] OR “Risk Factors”[tiab] OR “Risk Score”[tiab] OR “Risk Scores”[tiab] OR Risk[tiab] OR Risks[tiab] OR Predict[tiab] OR Predicts[tiab] OR Prediction[tiab] OR Validate[tiab] OR Validates[tiab] OR Model[tiab] OR Models[tiab] OR Models[tiab] OR Tool[tiab] OR Tools[tiab] OR Scale[tiab] OR Scales[tiab] OR Score[tiab] OR Scores[tiab] OR Predictor[tiab] OR Assessment[tiab] OR “Mathematical Model”[tiab] OR “Mathematical Models”[tiab] AND HIV[Mesh] OR HTLV-III[tiab] OR “Human Immunodeficiency Virus”[tiab] OR “Immunodeficiency Virus, Human”[tiab] OR “Immunodeficiency Viruses, Human”[tiab] OR “Virus, Human Immunodeficiency”[tiab] OR “Viruses, Human Immunodeficiency”[tiab] OR “Human Immunodeficiency Viruses”[tiab] OR “Human T Cell Lymphotropic Virus Type III”[tiab] OR “Human T-Cell Lymphotropic Virus Type III”[tiab] OR “Human T-Cell Leukemia Virus Type III”[tiab] OR “Human T Cell Leukemia Virus Type III”[tiab] OR “LAV-HTLV-III”[tiab] OR “Lymphadenopathy-Associated Virus”[tiab] OR “Lymphadenopathy Associated Virus”[tiab] OR “Lymphadenopathy-Associated Viruses”[tiab] OR “Viruses, Lymphadenopathy-Associated”[tiab] OR “Virus, Lymphadenopathy-Associated”[tiab] OR “Human T Lymphotropic Virus Type III”[tiab] OR “Human T-Lymphotropic Virus Type III”[tiab] OR “AIDS Virus”[tiab] OR “AIDS Viruses”[tiab] OR “Virus, AIDS”[tiab] OR “Viruses, AIDS”[tiab] OR “Acquired Immune Deficiency Syndrome Virus”[tiab] OR “Acquired Immunodeficiency Syndrome Virus”[tiab] OR HIV/AIDS[tiab] OR AIDS[tiab] AND "Sub-Saharan Africa"[tiab] OR "Sub Saharan Africa"[tiab] OR "Sub Sahara Africa"[tiab] OR "Sub-Sahara Africa"[tiab] OR SSA[tiab] OR “Central Africa”[tiab] OR Angola[tiab] OR Cameroon[tiab] OR “Central African Republic”[tiab] OR Chad[tiab] OR “Democratic Republic of the Congo”[tiab] OR DRC[tiab] OR “Equatorial Guinea”[tiab] OR Gabon[tiab] OR “Republic of the Congo”[tiab] OR “East Africa”[tiab] OR Burundi[tiab] OR Comoros[tiab] OR Djibouti[tiab] OR Eritrea[tiab] OR Ethiopia[tiab] OR Kenya[tiab] OR Madagascar[tiab] OR Malawi[tiab] OR Mauritius[tiab] OR Mozambique[tiab] OR Rwanda[tiab] OR Seychelles[tiab] OR Somalia[tiab] OR “South Sudan” [tiab] OR Sudan[tiab] OR Tanzania[tiab] OR Uganda[tiab] OR Zambia[tiab] OR Zimbabwe[tiab] OR “Southern Africa”[tiab] OR Botswana[tiab] OR Eswatini[tiab] OR Swaziland[tiab] OR Lesotho[tiab] OR Namibia[tiab] OR “South Africa”[tiab] OR “West Africa”[tiab] OR Benin[tiab] OR “Burkina Faso”[tiab] OR “Cabo Verde”[tiab] OR “Cape Verde”[tiab] OR “Côte d'Ivoire”[tiab] OR “Ivory Coast”[tiab] OR Gambia[tiab] OR Ghana[tiab] OR Guinea[tiab] OR “Guinea-Bissau”[tiab] OR Liberia[tiab] OR Mali[tiab] OR Mauritania[tiab] OR Niger[tiab] OR Nigeria[tiab] OR Senegal[tiab] OR “Sierra Leone”[tiab] OR Togo[tiab]

| **Search number** | **Query** | **Sort By** | **Filters** | **Search Details** | **Results** | **Time** | **Date** |
| --- | --- | --- | --- | --- | --- | --- | --- |
| 5 | ((("Cardiovascular Diseases"[Mesh] OR CVD[tiab] OR "Cardiovascular Disease"[tiab] OR "Disease, Cardiovascular"[tiab] OR "Cardiac Events"[tiab] OR "Cardiac Event"[tiab] OR "Event, Cardiac"[tiab] OR "Adverse Cardiac Event"[tiab] OR "Adverse Cardiac Events"[tiab] OR "Cardiac Event, Adverse"[tiab] OR "Cardiac Events, Adverse"[tiab] OR "Major Adverse Cardiac Events"[tiab] OR "Coronary Heart Disease"[tiab] OR CHD[tiab] OR "Ischemic Diseases"[tiab] OR "Coronary Artery Disease"[tiab] OR CAD[tiab] OR "Myocardial Infarction"[tiab] OR "Heart Attack"[tiab] OR "Angina Pectoris"[tiab] OR "Coronary Artery Spasm"[tiab] OR "Cerebrovascular Disease"[tiab] OR Stroke[tiab] OR Ischemic[tiab] OR "Transient Ischemic Attack"[tiab] OR TIA[tiab] OR "Cerebral Aneurysm"[tiab] OR "Heart Failure"[tiab] OR Cardiomyopathy[tiab] OR "Congestive Heart Failure"[tiab] OR Cardiomyopathy[tiab] OR Arrhythmias[tiab] OR "Heart Rhythm Disorders"[tiab] OR "Atrial Fibrillation"[tiab] OR AFib[tiab] OR Bradycardia[tiab] OR "Ventricular Tachycardia"[tiab] OR Fibrillation[tiab] OR "Peripheral Vascular Disease"[tiab] OR PVD[tiab] OR "Peripheral Artery Disease"[tiab] OR PAD[tiab] OR "Deep Vein Thrombosis"[tiab] OR DVT[tiab] OR "Pulmonary Embolism"[tiab] OR PE[tiab] OR "Varicose Veins"[tiab] OR "Valvular Heart Disease"[tiab] OR "Structural Heart Disease"[tiab] OR "Heart Valve Disease"[tiab] OR "Congenital Heart Disease"[tiab] OR "Congenital Heart Disease"[tiab] OR "Aortic Diseases"[tiab] OR "Pericardial Diseases"[tiab] OR "Aortic Aneurysm"[tiab] OR "Aortic Dissection"[tiab] OR Pericarditis[tiab] OR "Hypertensive Heart Diseases"[tiab] OR "Inflammatory Heart Diseases"[tiab] OR "Hypertensive Heart Disease"[tiab] OR Endocarditis[tiab] OR Myocarditis[tiab]) AND ("Risk Prediction"[tiab] OR "Risk Predictions"[tiab] OR "Risk Prediction Models"[tiab] OR "Risk Prediction Model"[tiab] OR "Risk Assessment"[tiab] OR "Risk Assessments"[tiab] OR "Risk Factor"[tiab] OR "Risk Factors"[tiab] OR "Risk Score"[tiab] OR "Risk Scores"[tiab] OR Risk[tiab] OR Risks[tiab] OR Predict[tiab] OR Predicts[tiab] OR Prediction[tiab] OR Validate[tiab] OR Validates[tiab] OR Model[tiab] OR Models[tiab] OR Models[tiab] OR Tool[tiab] OR Tools[tiab] OR Scale[tiab] OR Scales[tiab] OR Score[tiab] OR Scores[tiab] OR Predictor[tiab] OR Assessment[tiab] OR "Mathematical Model"[tiab] OR "Mathematical Models"[tiab])) AND (HIV[Mesh] OR HTLV-III[tiab] OR "Human Immunodeficiency Virus"[tiab] OR "Immunodeficiency Virus, Human"[tiab] OR "Immunodeficiency Viruses, Human"[tiab] OR "Virus, Human Immunodeficiency"[tiab] OR "Viruses, Human Immunodeficiency"[tiab] OR "Human Immunodeficiency Viruses"[tiab] OR "Human T Cell Lymphotropic Virus Type III"[tiab] OR "Human T-Cell Lymphotropic Virus Type III"[tiab] OR "Human T-Cell Leukemia Virus Type III"[tiab] OR "Human T Cell Leukemia Virus Type III"[tiab] OR "LAV-HTLV-III"[tiab] OR "Lymphadenopathy-Associated Virus"[tiab] OR "Lymphadenopathy Associated Virus"[tiab] OR "Lymphadenopathy-Associated Viruses"[tiab] OR "Viruses, Lymphadenopathy-Associated"[tiab] OR "Virus, Lymphadenopathy-Associated"[tiab] OR "Human T Lymphotropic Virus Type III"[tiab] OR "Human T-Lymphotropic Virus Type III"[tiab] OR "AIDS Virus"[tiab] OR "AIDS Viruses"[tiab] OR "Virus, AIDS"[tiab] OR "Viruses, AIDS"[tiab] OR "Acquired Immune Deficiency Syndrome Virus"[tiab] OR "Acquired Immunodeficiency Syndrome Virus"[tiab] OR HIV/AIDS[tiab] OR AIDS[tiab])) AND ("Sub-Saharan Africa"[tiab] OR "Sub Saharan Africa"[tiab] OR "Sub Sahara Africa"[tiab] OR "Sub-Sahara Africa"[tiab] OR SSA[tiab] OR "Central Africa"[tiab] OR Angola[tiab] OR Cameroon[tiab] OR "Central African Republic"[tiab] OR Chad[tiab] OR "Democratic Republic of the Congo"[tiab] OR DRC[tiab] OR "Equatorial Guinea"[tiab] OR Gabon[tiab] OR "Republic of the Congo"[tiab] OR "East Africa"[tiab] OR Burundi[tiab] OR Comoros[tiab] OR Djibouti[tiab] OR Eritrea[tiab] OR Ethiopia[tiab] OR Kenya[tiab] OR Madagascar[tiab] OR Malawi[tiab] OR Mauritius[tiab] OR Mozambique[tiab] OR Rwanda[tiab] OR Seychelles[tiab] OR Somalia[tiab] OR "South Sudan" [tiab] OR Sudan[tiab] OR Tanzania[tiab] OR Uganda[tiab] OR Zambia[tiab] OR Zimbabwe[tiab] OR "Southern Africa"[tiab] OR Botswana[tiab] OR Eswatini[tiab] OR Swaziland[tiab] OR Lesotho[tiab] OR Namibia[tiab] OR "South Africa"[tiab] OR "West Africa"[tiab] OR Benin[tiab] OR "Burkina Faso"[tiab] OR "Cabo Verde"[tiab] OR "Cape Verde"[tiab] OR "Côte d'Ivoire"[tiab] OR "Ivory Coast"[tiab] OR Gambia[tiab] OR Ghana[tiab] OR Guinea[tiab] OR "Guinea-Bissau"[tiab] OR Liberia[tiab] OR Mali[tiab] OR Mauritania[tiab] OR Niger[tiab] OR Nigeria[tiab] OR Senegal[tiab] OR "Sierra Leone"[tiab] OR Togo[tiab]) |  |  | ("Cardiovascular Diseases"[MeSH Terms] OR "CVD"[Title/Abstract] OR "Cardiovascular Disease"[Title/Abstract] OR "disease cardiovascular"[Title/Abstract] OR "Cardiac Events"[Title/Abstract] OR "Cardiac Event"[Title/Abstract] OR "event cardiac"[Title/Abstract] OR "Adverse Cardiac Event"[Title/Abstract] OR "Adverse Cardiac Events"[Title/Abstract] OR "cardiac events adverse"[Title/Abstract] OR "Major Adverse Cardiac Events"[Title/Abstract] OR "Coronary Heart Disease"[Title/Abstract] OR "CHD"[Title/Abstract] OR "Ischemic Diseases"[Title/Abstract] OR "Coronary Artery Disease"[Title/Abstract] OR "CAD"[Title/Abstract] OR "Myocardial Infarction"[Title/Abstract] OR "Heart Attack"[Title/Abstract] OR "Angina Pectoris"[Title/Abstract] OR "Coronary Artery Spasm"[Title/Abstract] OR "Cerebrovascular Disease"[Title/Abstract] OR "Stroke"[Title/Abstract] OR "Ischemic"[Title/Abstract] OR "Transient Ischemic Attack"[Title/Abstract] OR "TIA"[Title/Abstract] OR "Cerebral Aneurysm"[Title/Abstract] OR "Heart Failure"[Title/Abstract] OR "Cardiomyopathy"[Title/Abstract] OR "Congestive Heart Failure"[Title/Abstract] OR "Cardiomyopathy"[Title/Abstract] OR "Arrhythmias"[Title/Abstract] OR "Heart Rhythm Disorders"[Title/Abstract] OR "Atrial Fibrillation"[Title/Abstract] OR "AFib"[Title/Abstract] OR "Bradycardia"[Title/Abstract] OR "Ventricular Tachycardia"[Title/Abstract] OR "Fibrillation"[Title/Abstract] OR "Peripheral Vascular Disease"[Title/Abstract] OR "PVD"[Title/Abstract] OR "Peripheral Artery Disease"[Title/Abstract] OR "PAD"[Title/Abstract] OR "Deep Vein Thrombosis"[Title/Abstract] OR "DVT"[Title/Abstract] OR "Pulmonary Embolism"[Title/Abstract] OR "PE"[Title/Abstract] OR "Varicose Veins"[Title/Abstract] OR "Valvular Heart Disease"[Title/Abstract] OR "Structural Heart Disease"[Title/Abstract] OR "Heart Valve Disease"[Title/Abstract] OR "Congenital Heart Disease"[Title/Abstract] OR "Congenital Heart Disease"[Title/Abstract] OR "Aortic Diseases"[Title/Abstract] OR "Pericardial Diseases"[Title/Abstract] OR "Aortic Aneurysm"[Title/Abstract] OR "Aortic Dissection"[Title/Abstract] OR "Pericarditis"[Title/Abstract] OR "Hypertensive Heart Diseases"[Title/Abstract] OR "Inflammatory Heart Diseases"[Title/Abstract] OR "Hypertensive Heart Disease"[Title/Abstract] OR "Endocarditis"[Title/Abstract] OR "Myocarditis"[Title/Abstract]) AND ("Risk Prediction"[Title/Abstract] OR "Risk Predictions"[Title/Abstract] OR "Risk Prediction Models"[Title/Abstract] OR "Risk Prediction Model"[Title/Abstract] OR "Risk Assessment"[Title/Abstract] OR "Risk Assessments"[Title/Abstract] OR "Risk Factor"[Title/Abstract] OR "Risk Factors"[Title/Abstract] OR "Risk Score"[Title/Abstract] OR "Risk Scores"[Title/Abstract] OR "Risk"[Title/Abstract] OR "Risks"[Title/Abstract] OR "Predict"[Title/Abstract] OR "Predicts"[Title/Abstract] OR "Prediction"[Title/Abstract] OR "Validate"[Title/Abstract] OR "Validates"[Title/Abstract] OR "Model"[Title/Abstract] OR "Models"[Title/Abstract] OR "Models"[Title/Abstract] OR "Tool"[Title/Abstract] OR "Tools"[Title/Abstract] OR "Scale"[Title/Abstract] OR "Scales"[Title/Abstract] OR "Score"[Title/Abstract] OR "Scores"[Title/Abstract] OR "Predictor"[Title/Abstract] OR "Assessment"[Title/Abstract] OR "Mathematical Model"[Title/Abstract] OR "Mathematical Models"[Title/Abstract]) AND ("HIV"[MeSH Terms] OR "HTLV-III"[Title/Abstract] OR "Human Immunodeficiency Virus"[Title/Abstract] OR "immunodeficiency virus human"[Title/Abstract] OR "immunodeficiency viruses human"[Title/Abstract] OR "virus human immunodeficiency"[Title/Abstract] OR "viruses human immunodeficiency"[Title/Abstract] OR "Human Immunodeficiency Viruses"[Title/Abstract] OR "human t cell lymphotropic virus type iii"[Title/Abstract] OR "human t cell lymphotropic virus type iii"[Title/Abstract] OR "human t cell leukemia virus type iii"[Title/Abstract] OR "human t cell leukemia virus type iii"[Title/Abstract] OR "LAV-HTLV-III"[Title/Abstract] OR "lymphadenopathy associated virus"[Title/Abstract] OR "lymphadenopathy associated virus"[Title/Abstract] OR "viruses lymphadenopathy associated"[Title/Abstract] OR "virus lymphadenopathy associated"[Title/Abstract] OR "human t lymphotropic virus type iii"[Title/Abstract] OR "human t lymphotropic virus type iii"[Title/Abstract] OR "AIDS Virus"[Title/Abstract] OR "AIDS Viruses"[Title/Abstract] OR "virus aids"[Title/Abstract] OR "viruses aids"[Title/Abstract] OR "Acquired Immune Deficiency Syndrome Virus"[Title/Abstract] OR "Acquired Immunodeficiency Syndrome Virus"[Title/Abstract] OR "hiv aids"[Title/Abstract] OR "AIDS"[Title/Abstract]) AND ("sub saharan africa"[Title/Abstract] OR "sub saharan africa"[Title/Abstract] OR "sub sahara africa"[Title/Abstract] OR "sub sahara africa"[Title/Abstract] OR "SSA"[Title/Abstract] OR "Central Africa"[Title/Abstract] OR "Angola"[Title/Abstract] OR "Cameroon"[Title/Abstract] OR "Central African Republic"[Title/Abstract] OR "Chad"[Title/Abstract] OR "Democratic Republic of the Congo"[Title/Abstract] OR "DRC"[Title/Abstract] OR "Equatorial Guinea"[Title/Abstract] OR "Gabon"[Title/Abstract] OR "Republic of the Congo"[Title/Abstract] OR "East Africa"[Title/Abstract] OR "Burundi"[Title/Abstract] OR "Comoros"[Title/Abstract] OR "Djibouti"[Title/Abstract] OR "Eritrea"[Title/Abstract] OR "Ethiopia"[Title/Abstract] OR "Kenya"[Title/Abstract] OR "Madagascar"[Title/Abstract] OR "Malawi"[Title/Abstract] OR "Mauritius"[Title/Abstract] OR "Mozambique"[Title/Abstract] OR "Rwanda"[Title/Abstract] OR "Seychelles"[Title/Abstract] OR "Somalia"[Title/Abstract] OR "South Sudan"[Title/Abstract] OR "Sudan"[Title/Abstract] OR "Tanzania"[Title/Abstract] OR "Uganda"[Title/Abstract] OR "Zambia"[Title/Abstract] OR "Zimbabwe"[Title/Abstract] OR "Southern Africa"[Title/Abstract] OR "Botswana"[Title/Abstract] OR "Eswatini"[Title/Abstract] OR "Swaziland"[Title/Abstract] OR "Lesotho"[Title/Abstract] OR "Namibia"[Title/Abstract] OR "South Africa"[Title/Abstract] OR "West Africa"[Title/Abstract] OR "Benin"[Title/Abstract] OR "Burkina Faso"[Title/Abstract] OR "Cabo Verde"[Title/Abstract] OR "Cape Verde"[Title/Abstract] OR "Cote d'Ivoire"[Title/Abstract] OR "Ivory Coast"[Title/Abstract] OR "Gambia"[Title/Abstract] OR "Ghana"[Title/Abstract] OR "Guinea"[Title/Abstract] OR "Guinea-Bissau"[Title/Abstract] OR "Liberia"[Title/Abstract] OR "Mali"[Title/Abstract] OR "Mauritania"[Title/Abstract] OR "Niger"[Title/Abstract] OR "Nigeria"[Title/Abstract] OR "Senegal"[Title/Abstract] OR "Sierra Leone"[Title/Abstract] OR "Togo"[Title/Abstract]) | 409 | 09:46:47 | 2026/06/03 |
| 4 | "Sub-Saharan Africa"[tiab] OR "Sub Saharan Africa"[tiab] OR "Sub Sahara Africa"[tiab] OR "Sub-Sahara Africa"[tiab] OR SSA[tiab] OR "Central Africa"[tiab] OR Angola[tiab] OR Cameroon[tiab] OR "Central African Republic"[tiab] OR Chad[tiab] OR "Democratic Republic of the Congo"[tiab] OR DRC[tiab] OR "Equatorial Guinea"[tiab] OR Gabon[tiab] OR "Republic of the Congo"[tiab] OR "East Africa"[tiab] OR Burundi[tiab] OR Comoros[tiab] OR Djibouti[tiab] OR Eritrea[tiab] OR Ethiopia[tiab] OR Kenya[tiab] OR Madagascar[tiab] OR Malawi[tiab] OR Mauritius[tiab] OR Mozambique[tiab] OR Rwanda[tiab] OR Seychelles[tiab] OR Somalia[tiab] OR "South Sudan" [tiab] OR Sudan[tiab] OR Tanzania[tiab] OR Uganda[tiab] OR Zambia[tiab] OR Zimbabwe[tiab] OR "Southern Africa"[tiab] OR Botswana[tiab] OR Eswatini[tiab] OR Swaziland[tiab] OR Lesotho[tiab] OR Namibia[tiab] OR "South Africa"[tiab] OR "West Africa"[tiab] OR Benin[tiab] OR "Burkina Faso"[tiab] OR "Cabo Verde"[tiab] OR "Cape Verde"[tiab] OR "Côte d'Ivoire"[tiab] OR "Ivory Coast"[tiab] OR Gambia[tiab] OR Ghana[tiab] OR Guinea[tiab] OR "Guinea-Bissau"[tiab] OR Liberia[tiab] OR Mali[tiab] OR Mauritania[tiab] OR Niger[tiab] OR Nigeria[tiab] OR Senegal[tiab] OR "Sierra Leone"[tiab] OR Togo[tiab] |  |  | "sub saharan africa"[Title/Abstract] OR "sub saharan africa"[Title/Abstract] OR "sub sahara africa"[Title/Abstract] OR "sub sahara africa"[Title/Abstract] OR "SSA"[Title/Abstract] OR "Central Africa"[Title/Abstract] OR "Angola"[Title/Abstract] OR "Cameroon"[Title/Abstract] OR "Central African Republic"[Title/Abstract] OR "Chad"[Title/Abstract] OR "Democratic Republic of the Congo"[Title/Abstract] OR "DRC"[Title/Abstract] OR "Equatorial Guinea"[Title/Abstract] OR "Gabon"[Title/Abstract] OR "Republic of the Congo"[Title/Abstract] OR "East Africa"[Title/Abstract] OR "Burundi"[Title/Abstract] OR "Comoros"[Title/Abstract] OR "Djibouti"[Title/Abstract] OR "Eritrea"[Title/Abstract] OR "Ethiopia"[Title/Abstract] OR "Kenya"[Title/Abstract] OR "Madagascar"[Title/Abstract] OR "Malawi"[Title/Abstract] OR "Mauritius"[Title/Abstract] OR "Mozambique"[Title/Abstract] OR "Rwanda"[Title/Abstract] OR "Seychelles"[Title/Abstract] OR "Somalia"[Title/Abstract] OR "South Sudan"[Title/Abstract] OR "Sudan"[Title/Abstract] OR "Tanzania"[Title/Abstract] OR "Uganda"[Title/Abstract] OR "Zambia"[Title/Abstract] OR "Zimbabwe"[Title/Abstract] OR "Southern Africa"[Title/Abstract] OR "Botswana"[Title/Abstract] OR "Eswatini"[Title/Abstract] OR "Swaziland"[Title/Abstract] OR "Lesotho"[Title/Abstract] OR "Namibia"[Title/Abstract] OR "South Africa"[Title/Abstract] OR "West Africa"[Title/Abstract] OR "Benin"[Title/Abstract] OR "Burkina Faso"[Title/Abstract] OR "Cabo Verde"[Title/Abstract] OR "Cape Verde"[Title/Abstract] OR "Cote d'Ivoire"[Title/Abstract] OR "Ivory Coast"[Title/Abstract] OR "Gambia"[Title/Abstract] OR "Ghana"[Title/Abstract] OR "Guinea"[Title/Abstract] OR "Guinea-Bissau"[Title/Abstract] OR "Liberia"[Title/Abstract] OR "Mali"[Title/Abstract] OR "Mauritania"[Title/Abstract] OR "Niger"[Title/Abstract] OR "Nigeria"[Title/Abstract] OR "Senegal"[Title/Abstract] OR "Sierra Leone"[Title/Abstract] OR "Togo"[Title/Abstract] | 493,280 | 09:46:21 | 2026/06/03 |
| 3 | HIV[Mesh] OR HTLV-III[tiab] OR "Human Immunodeficiency Virus"[tiab] OR "Immunodeficiency Virus, Human"[tiab] OR "Immunodeficiency Viruses, Human"[tiab] OR "Virus, Human Immunodeficiency"[tiab] OR "Viruses, Human Immunodeficiency"[tiab] OR "Human Immunodeficiency Viruses"[tiab] OR "Human T Cell Lymphotropic Virus Type III"[tiab] OR "Human T-Cell Lymphotropic Virus Type III"[tiab] OR "Human T-Cell Leukemia Virus Type III"[tiab] OR "Human T Cell Leukemia Virus Type III"[tiab] OR "LAV-HTLV-III"[tiab] OR "Lymphadenopathy-Associated Virus"[tiab] OR "Lymphadenopathy Associated Virus"[tiab] OR "Lymphadenopathy-Associated Viruses"[tiab] OR "Viruses, Lymphadenopathy-Associated"[tiab] OR "Virus, Lymphadenopathy-Associated"[tiab] OR "Human T Lymphotropic Virus Type III"[tiab] OR "Human T-Lymphotropic Virus Type III"[tiab] OR "AIDS Virus"[tiab] OR "AIDS Viruses"[tiab] OR "Virus, AIDS"[tiab] OR "Viruses, AIDS"[tiab] OR "Acquired Immune Deficiency Syndrome Virus"[tiab] OR "Acquired Immunodeficiency Syndrome Virus"[tiab] OR HIV/AIDS[tiab] OR AIDS[tiab] |  |  | "HIV"[MeSH Terms] OR "HTLV-III"[Title/Abstract] OR "Human Immunodeficiency Virus"[Title/Abstract] OR "immunodeficiency virus human"[Title/Abstract] OR "immunodeficiency viruses human"[Title/Abstract] OR "virus human immunodeficiency"[Title/Abstract] OR "viruses human immunodeficiency"[Title/Abstract] OR "Human Immunodeficiency Viruses"[Title/Abstract] OR "human t cell lymphotropic virus type iii"[Title/Abstract] OR "human t cell lymphotropic virus type iii"[Title/Abstract] OR "human t cell leukemia virus type iii"[Title/Abstract] OR "human t cell leukemia virus type iii"[Title/Abstract] OR "LAV-HTLV-III"[Title/Abstract] OR "lymphadenopathy associated virus"[Title/Abstract] OR "lymphadenopathy associated virus"[Title/Abstract] OR "viruses lymphadenopathy associated"[Title/Abstract] OR "virus lymphadenopathy associated"[Title/Abstract] OR "human t lymphotropic virus type iii"[Title/Abstract] OR "human t lymphotropic virus type iii"[Title/Abstract] OR "AIDS Virus"[Title/Abstract] OR "AIDS Viruses"[Title/Abstract] OR "virus aids"[Title/Abstract] OR "viruses aids"[Title/Abstract] OR "Acquired Immune Deficiency Syndrome Virus"[Title/Abstract] OR "Acquired Immunodeficiency Syndrome Virus"[Title/Abstract] OR "hiv aids"[Title/Abstract] OR "AIDS"[Title/Abstract] | 334,340 | 09:45:56 | 2026/06/03 |
| 2 | "Risk Prediction"[tiab] OR "Risk Predictions"[tiab] OR "Risk Prediction Models"[tiab] OR "Risk Prediction Model"[tiab] OR "Risk Assessment"[tiab] OR "Risk Assessments"[tiab] OR "Risk Factor"[tiab] OR "Risk Factors"[tiab] OR "Risk Score"[tiab] OR "Risk Scores"[tiab] OR Risk[tiab] OR Risks[tiab] OR Predict[tiab] OR Predicts[tiab] OR Prediction[tiab] OR Validate[tiab] OR Validates[tiab] OR Model[tiab] OR Models[tiab] OR Models[tiab] OR Tool[tiab] OR Tools[tiab] OR Scale[tiab] OR Scales[tiab] OR Score[tiab] OR Scores[tiab] OR Predictor[tiab] OR Assessment[tiab] OR "Mathematical Model"[tiab] OR "Mathematical Models"[tiab] |  |  | "Risk Prediction"[Title/Abstract] OR "Risk Predictions"[Title/Abstract] OR "Risk Prediction Models"[Title/Abstract] OR "Risk Prediction Model"[Title/Abstract] OR "Risk Assessment"[Title/Abstract] OR "Risk Assessments"[Title/Abstract] OR "Risk Factor"[Title/Abstract] OR "Risk Factors"[Title/Abstract] OR "Risk Score"[Title/Abstract] OR "Risk Scores"[Title/Abstract] OR "Risk"[Title/Abstract] OR "Risks"[Title/Abstract] OR "Predict"[Title/Abstract] OR "Predicts"[Title/Abstract] OR "Prediction"[Title/Abstract] OR "Validate"[Title/Abstract] OR "Validates"[Title/Abstract] OR "Model"[Title/Abstract] OR "Models"[Title/Abstract] OR "Models"[Title/Abstract] OR "Tool"[Title/Abstract] OR "Tools"[Title/Abstract] OR "Scale"[Title/Abstract] OR "Scales"[Title/Abstract] OR "Score"[Title/Abstract] OR "Scores"[Title/Abstract] OR "Predictor"[Title/Abstract] OR "Assessment"[Title/Abstract] OR "Mathematical Model"[Title/Abstract] OR "Mathematical Models"[Title/Abstract] | 11,055,779 | 09:45:34 | 2026/06/03 |
| 1 | "Cardiovascular Diseases"[Mesh] OR CVD[tiab] OR "Cardiovascular Disease"[tiab] OR "Disease, Cardiovascular"[tiab] OR "Cardiac Events"[tiab] OR "Cardiac Event"[tiab] OR "Event, Cardiac"[tiab] OR "Adverse Cardiac Event"[tiab] OR "Adverse Cardiac Events"[tiab] OR "Cardiac Event, Adverse"[tiab] OR "Cardiac Events, Adverse"[tiab] OR "Major Adverse Cardiac Events"[tiab] OR "Coronary Heart Disease"[tiab] OR CHD[tiab] OR "Ischemic Diseases"[tiab] OR "Coronary Artery Disease"[tiab] OR CAD[tiab] OR "Myocardial Infarction"[tiab] OR "Heart Attack"[tiab] OR "Angina Pectoris"[tiab] OR "Coronary Artery Spasm"[tiab] OR "Cerebrovascular Disease"[tiab] OR Stroke[tiab] OR Ischemic[tiab] OR "Transient Ischemic Attack"[tiab] OR TIA[tiab] OR "Cerebral Aneurysm"[tiab] OR "Heart Failure"[tiab] OR Cardiomyopathy[tiab] OR "Congestive Heart Failure"[tiab] OR Cardiomyopathy[tiab] OR Arrhythmias[tiab] OR "Heart Rhythm Disorders"[tiab] OR "Atrial Fibrillation"[tiab] OR AFib[tiab] OR Bradycardia[tiab] OR "Ventricular Tachycardia"[tiab] OR Fibrillation[tiab] OR "Peripheral Vascular Disease"[tiab] OR PVD[tiab] OR "Peripheral Artery Disease"[tiab] OR PAD[tiab] OR "Deep Vein Thrombosis"[tiab] OR DVT[tiab] OR "Pulmonary Embolism"[tiab] OR PE[tiab] OR "Varicose Veins"[tiab] OR "Valvular Heart Disease"[tiab] OR "Structural Heart Disease"[tiab] OR "Heart Valve Disease"[tiab] OR "Congenital Heart Disease"[tiab] OR "Congenital Heart Disease"[tiab] OR "Aortic Diseases"[tiab] OR "Pericardial Diseases"[tiab] OR "Aortic Aneurysm"[tiab] OR "Aortic Dissection"[tiab] OR Pericarditis[tiab] OR "Hypertensive Heart Diseases"[tiab] OR "Inflammatory Heart Diseases"[tiab] OR "Hypertensive Heart Disease"[tiab] OR Endocarditis[tiab] OR Myocarditis[tiab] |  |  | "Cardiovascular Diseases"[MeSH Terms] OR "CVD"[Title/Abstract] OR "Cardiovascular Disease"[Title/Abstract] OR "disease cardiovascular"[Title/Abstract] OR "Cardiac Events"[Title/Abstract] OR "Cardiac Event"[Title/Abstract] OR "event cardiac"[Title/Abstract] OR "Adverse Cardiac Event"[Title/Abstract] OR "Adverse Cardiac Events"[Title/Abstract] OR "cardiac events adverse"[Title/Abstract] OR "Major Adverse Cardiac Events"[Title/Abstract] OR "Coronary Heart Disease"[Title/Abstract] OR "CHD"[Title/Abstract] OR "Ischemic Diseases"[Title/Abstract] OR "Coronary Artery Disease"[Title/Abstract] OR "CAD"[Title/Abstract] OR "Myocardial Infarction"[Title/Abstract] OR "Heart Attack"[Title/Abstract] OR "Angina Pectoris"[Title/Abstract] OR "Coronary Artery Spasm"[Title/Abstract] OR "Cerebrovascular Disease"[Title/Abstract] OR "Stroke"[Title/Abstract] OR "Ischemic"[Title/Abstract] OR "Transient Ischemic Attack"[Title/Abstract] OR "TIA"[Title/Abstract] OR "Cerebral Aneurysm"[Title/Abstract] OR "Heart Failure"[Title/Abstract] OR "Cardiomyopathy"[Title/Abstract] OR "Congestive Heart Failure"[Title/Abstract] OR "Cardiomyopathy"[Title/Abstract] OR "Arrhythmias"[Title/Abstract] OR "Heart Rhythm Disorders"[Title/Abstract] OR "Atrial Fibrillation"[Title/Abstract] OR "AFib"[Title/Abstract] OR "Bradycardia"[Title/Abstract] OR "Ventricular Tachycardia"[Title/Abstract] OR "Fibrillation"[Title/Abstract] OR "Peripheral Vascular Disease"[Title/Abstract] OR "PVD"[Title/Abstract] OR "Peripheral Artery Disease"[Title/Abstract] OR "PAD"[Title/Abstract] OR "Deep Vein Thrombosis"[Title/Abstract] OR "DVT"[Title/Abstract] OR "Pulmonary Embolism"[Title/Abstract] OR "PE"[Title/Abstract] OR "Varicose Veins"[Title/Abstract] OR "Valvular Heart Disease"[Title/Abstract] OR "Structural Heart Disease"[Title/Abstract] OR "Heart Valve Disease"[Title/Abstract] OR "Congenital Heart Disease"[Title/Abstract] OR "Congenital Heart Disease"[Title/Abstract] OR "Aortic Diseases"[Title/Abstract] OR "Pericardial Diseases"[Title/Abstract] OR "Aortic Aneurysm"[Title/Abstract] OR "Aortic Dissection"[Title/Abstract] OR "Pericarditis"[Title/Abstract] OR "Hypertensive Heart Diseases"[Title/Abstract] OR "Inflammatory Heart Diseases"[Title/Abstract] OR "Hypertensive Heart Disease"[Title/Abstract] OR "Endocarditis"[Title/Abstract] OR "Myocarditis"[Title/Abstract] | 3,589,545 | 09:45:09 | 2026/06/03 |
